# Modelling the impact of salt reduction policies on hypertension in Portugal

**DOI:** 10.1093/eurpub/ckac129.494

**Published:** 2022-10-25

**Authors:** N Lorenzo i Sunyer, M Severo, MJ Gregório, C Lopes

**Affiliations:** 1 EPIUnit/ITR- Institute Public Health, University of Porto, Porto, Portugal; 2 Institute of Biomedical Sciences Abel Salazar, University of Porto, Porto, Portugal; 3 Food and Nutrition Science Faculty, University of Porto, Porto, Portugal; 4 Faculty of Medicine, University of Porto, Port, Portugal

## Abstract

Salt reduction policies are identified as effective; however, the assessment of their impact in European countries has been underexplored. The aim of this study is to assess the impact of salt reduction policies on hypertension in Portugal, to ultimately guide future policies. Based on national data, the top five foods contributing to salt intake, their salt content, and daily consumption were determined. Food reformulation included current policies and proposed targets for future agreements. Food availability trend from the main contributors was estimated from national food balance sheets. For this study, we considered five counterfactual scenarios (CF) to estimate salt intake variation: (1) reduction of salt content of foods targeted by current policies, while assuming stable consumption of the primary contributors; (2) proposal to extend CF 1 to other food categories; (3) change in the principal contributor's consumption based on the trend, assuming stable salt content; (4) combination of CF 2 and 3; (5) CF 4 and assuming a reduction of “1 pinch of salt”. Relative risk was estimated from regression coefficients to then calculate the potential impact fraction (PIF) and ultimately provide the hypertension cases prevented per year for each CF. The change in salt intake expected by each CF is -11.16%, -13.57%, +0.12%, -13.40% and 23,99%, respectively. For each CF, PIF and hypertension cases avoided per year was, as follows (mean (95%CI)): 9.46% and 46401 (44201;48925) for the first; 14.05% and 68921 (66149;72025) for the second; -1.24% and -6099 (-7636;-4763) for the third; 13.2% and 65125 (62286;67892) for the fourth; and, 15.58% and 76397 (73208;80021) for the last. This study suggests that if the salt content of main contributors was not reduced, its increasing food trend consumption might result in higher incidence of hypertension in Portugal. However, by combining current policies with targets on additional foods, more than 60,000 cases could be avoided annually.

**Key messages:**

• Salt content reduction policies are necessary if we want to reduce the incidence of hypertension among the Portuguese population.

• Assessment of the impact of salt reduction policies is crucial if we want to choose those that have a stronger impact on the health of our population.

